# Transient Neuropathic Pain and Sensory Hypersensitivity Following Single-Pulse Transcranial Magnetic Stimulation: A Case Report

**DOI:** 10.7759/cureus.99340

**Published:** 2025-12-15

**Authors:** Keiichiro Aoki, Yu Nakajima

**Affiliations:** 1 Division of Occupational Therapy, Department of Rehabilitation, School of Nursing and Rehabilitation Sciences, Showa University, Yokohamashi, JPN; 2 Rehabilitation, C-BRIGHT Inc., Tokyo, JPN

**Keywords:** case report, hyperesthesia, mirogabalin, neuropathic pain, sensory modulation, single-pulse tms, tactile allodynia, tens, vibration therapy

## Abstract

This case report describes a rare instance of transient neuropathic pain and thermal hypersensitivity in a healthy adult following low-intensity single-pulse transcranial magnetic stimulation (TMS). Single-pulse TMS is generally regarded as safe, with only brief and mild sensory effects typically reported. In this case, subtle discomfort in the contralateral hand immediately after stimulation progressed over several days to tingling, warmth, and thermal hyperesthesia. The neurological examination and MRI revealed no structural abnormalities. Symptoms gradually improved with pharmacological treatment using mirogabalin, followed by non-pharmacological interventions, including vibration therapy, transcutaneous electrical nerve stimulation (TENS), and mirror therapy. These interventions provided short-term symptomatic relief and supported functional recovery. Complete resolution occurred within approximately two months. This report highlights that even low-intensity single-pulse TMS can, in rare situations, induce transient neuropathic-like sensory disturbances. Recognizing such presentations may help clinicians provide appropriate reassurance, monitor symptom progression, and apply targeted therapeutic strategies when unexpected sensory effects arise after TMS.

## Introduction

Transcranial magnetic stimulation (TMS) is a well-established, non-invasive method for assessing cortical excitability through brief magnetic pulses delivered to the scalp. Single-pulse TMS is widely used in neuroscience research because it provides direct evaluation of corticospinal excitability with minimal physiological burden, and its safety profile is strongly supported by international guidelines [1]. In contrast to repetitive TMS (rTMS), which has therapeutic applications in depression, neuropathic pain, migraine, and post-stroke rehabilitation [2], single-pulse stimulation is generally associated with only mild and transient sensations such as scalp tingling or headache [1,3,4,5]. Prolonged sensory disturbances after single-pulse TMS are exceedingly rare, particularly in healthy individuals. In this case, single-pulse TMS was administered as part of an experiential familiarization procedure following the acquisition of new research equipment. Such preliminary exposure is commonly performed to confirm device functionality, understand stimulation characteristics, and ensure user safety and tolerance before employing the equipment in formal research or clinical applications.

We describe an unusual case of transient neuropathic pain and thermal hypersensitivity following low-intensity single-pulse TMS in a healthy adult. The clinical course highlights the need to recognize uncommon sensory reactions and to offer appropriate monitoring and reassurance.

## Case presentation

A healthy right-handed male in his late 30s participated as a volunteer in a motor-mapping session. Approximately 10 single monophasic pulses were applied over the left primary motor cortex (C3) using a MagPro stimulator (MagVenture, Farum, Denmark) at 20-50% maximum stimulator output (MSO). During stimulation, he experienced a subtle but clearly identifiable discomfort in the contralateral hand. Although initially mild, this altered sensation did not fully dissipate following the session.

**Figure 1:**
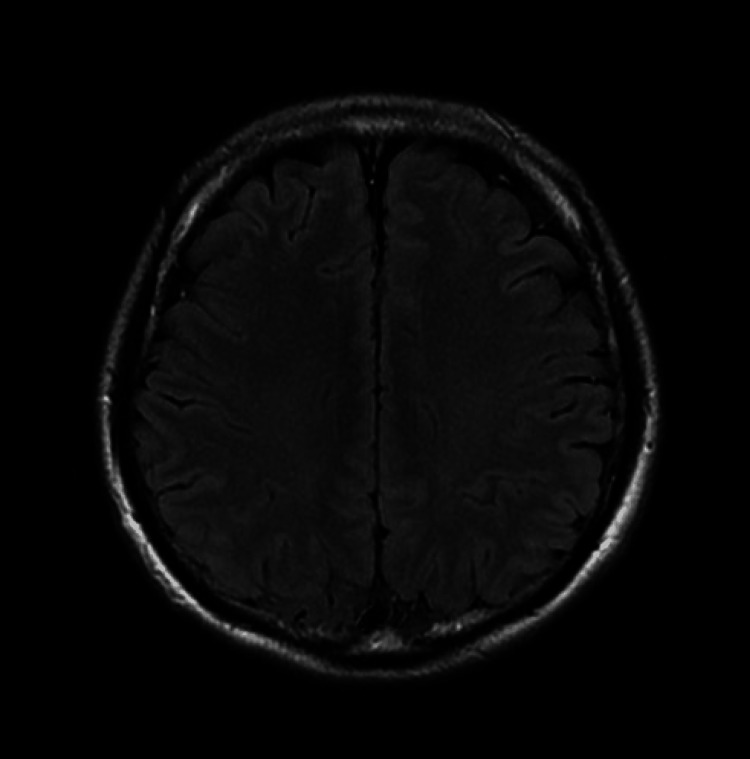
Axial FLAIR MRI obtained on post-stimulation day 17 The image demonstrates no structural abnormalities in the cerebral cortex or subcortical white matter FLAIR: fluid-attenuated inversion recovery; MRI: magnetic resonance imaging

Post-stimulation day one, the participant developed a more prominent tingling sensation in digits II-V of the right hand, accompanied by intermittent warmth and mild nocturnal discomfort. Cooling provided temporary relief. Symptoms fluctuated over the next several days, and on day five, a neurological evaluation suggested thermal hyperesthesia consistent with neuropathic hypersensitivity. Motor strength, reflexes, coordination, and light-touch sensitivity remained normal. No structural abnormalities were identified on MRI on day 17 (Figure 1).

Mirogabalin 5 mg twice daily was initiated on day six, resulting in gradual improvement with a reduction in Visual Analog Scale (VAS) from 2 to approximately 1. By day 30, residual symptoms persisted but were modest and stable. On the same day, the participant began a structured rehabilitation program at a self-paid facility, including guided vibration therapy, transcutaneous electrical nerve stimulation (TENS), and mirror therapy. These interventions produced immediate but transient reductions in symptom severity and improved tolerance to daily hand use.

On day 33, the primary physician advised that medication discontinuation was acceptable, and mirogabalin was stopped without symptom exacerbation (VAS ~1). Over the following two weeks, symptom intensity fluctuated mildly with activity (day 35: VAS 1; day 42: VAS 0.5; day 50: VAS 0.5), while the participant continued supervised rehabilitation and daily home-based exercises using rented equipment under remote guidance. By day 58, the rehabilitation program was completed, and all sensory symptoms had resolved (VAS 0). On day 68, follow-up assessment confirmed stable recovery without residual deficits, and the participant was transitioned to observation only. He reported full return to normal daily, occupational, and recreational activities without further sensory disturbances (Table 1).

**Table 1:** Clinical course and symptom progression following single-pulse TMS TMS: transcranial magnetic stimulation; VAS: Visual Analog Scale; MRI: magnetic resonance imaging; TENS: transcutaneous electrical nerve stimulation

Post-stimulation day	Clinical course	VAS (0-10)	Management/events	Remarks
0	Subtle discomfort in the right hand immediately after single-pulse TMS	2	Observation	Altered sensation persisted after the session
1	Tingling digits II–V; intermittent warmth; relieved by cooling	2	Cooling	Nocturnal discomfort; mild anxiety
5	Thermal hyperesthesia identified; neuropathic hypersensitivity suspected	2	Neurological examination	Motor/reflex/coordination intact
6	Mirogabalin initiated	2 → 1	Pharmacologic therapy	Rapid symptomatic improvement
17	MRI performed; no abnormalities	1	MRI	No structural lesions
30	Structured rehabilitation program initiated	1	Rehab (vibration therapy, TENS, mirror therapy)	Immediate but transient relief
33	Medication discontinued without worsening	1	Physician follow-up	Stable symptoms
35	Mild fluctuation with activity	1	Home-based exercises	Improved tolerance to hand use
42	Mild residual sensations only	0.5	Continued rehab	Stable improvement
50	Residual sensory discomfort with fatigue/overuse	0.5	Rehab + home program	No functional impact
58	Rehabilitation completed	0	Rehab completion	Full functional recovery
68	Follow-up only; no residual symptoms	0	Outpatient evaluation	No further intervention required

## Discussion

This case illustrates an uncommon sensory complication following low-intensity single-pulse TMS in a healthy adult. While transient scalp discomfort and mild headache are well-recognized benign effects [1,3,4], neuropathic-like symptoms persisting beyond the stimulation period are exceptionally rare [6]. The temporal evolution - immediate subtle discomfort followed hours later by tingling, warmth, and thermal hyperesthesia - suggests contributions from both early neurophysiological responses and delayed modulatory processes.

Several mechanisms may account for the presentation. Peripheral sensitization is plausible: indirect activation of cutaneous or muscle afferents by TMS-induced currents may lower the threshold for ectopic firing [6]. Central sensitization, involving enhanced processing within spinal or supraspinal nociceptive pathways, may also amplify sensory inputs [7]. Modulation of somatosensory and pain-related cortical networks may further intensify thermal and tactile perception despite normal MRI findings [8]. Psychophysiological factors likely contributed as well. Symptom exacerbation during periods of heightened attention or anxiety aligns with models of cognitive-emotional amplification, in which interoceptive focus and anxiety increase the salience of bodily sensations [9].

Therapeutic responses also provide clues. The beneficial effects of mirogabalin suggest involvement of sensitized nociceptive pathways rather than purely psychogenic mechanisms. Non-pharmacologic interventions yielded additional insights. TENS and vibration therapy provided immediate but transient relief consistent with gate-control mechanisms [10], while mirror therapy may have modulated maladaptive cortical representations and somatosensory processing [11]. From a safety perspective, although single-pulse TMS is regarded as extremely low risk [1,3,5], this case demonstrates that rare, transient neuropathic-like sensory disturbances can occur [6]. Whether this reflects individual susceptibility - baseline anxiety, sensory hypervigilance, or distinct cortical excitability profiles - remains uncertain.

Limitations of this report include the absence of electrophysiological testing and the use of multiple sequential interventions, preventing causal separation. Nonetheless, the temporal association with stimulation, hemispheric specificity, and targeted treatment response support a plausible link between TMS and the observed symptoms. Practically, clinicians should recognize that even mild sensory complaints after TMS may reflect transient alterations in sensory processing. A multimodal strategy - including pharmacologic agents, sensory-modulatory rehabilitation, graded activity, and psychological reassurance - may facilitate recovery and prevent chronicity.

## Conclusions

This report describes a rare instance of transient neuropathic pain and thermal hypersensitivity following low-intensity single-pulse TMS in a healthy adult. Although symptoms persisted for several weeks, they were fully reversed with a combination of pharmacologic therapy, sensory-modulation techniques, and structured rehabilitation. Clinicians should remain mindful of the potential for atypical sensory reactions and offer appropriate reassurance, monitoring, and individualized management when they arise.
